# Supplementary material for: Self-Reported Non-Celiac Wheat Sensitivity and Other Food Sensitivities in Patients with Primary Sjögren’s Syndrome
Source: Nutrients. 2025 Oct 8;17(19):3172. doi: 10.3390/nu17193172 (PMC12525645; doi:10.3390/nu17193172)
Supplement: Supplementary file 1 [file nutrients-17-03172-s001.zip › Supplementary File S1 - Methods - 11_09_2025.pdf]

Supplementary File S1.

Questionnaire on the prevalence of self-reported NCWS and/or other food hypersensitivities

**PART 1: GENERAL SECTION**
1.1 Age (years)
1.2 Gender<br>1. Male<br>2. Female
1.3 Ethnicity<br>1. Caucasian<br>2. African<br>3. Asian<br>4. Middle Eastern
1.4 Marital status<br>1. Single<br>2. Married<br>3. Divorced<br>4. Widowed
1.5 Education level<br>1. None<br>2. Elementary school diploma<br>3. Middle school diploma<br>4. High school diploma<br>5. University degree
1.6 Employment status<br>1. Employee<br>2. Freelance/Professional<br>3. Laborer/unskilled worker/craftsperson<br>4. Unemployed<br>5. Looking for work<br>6. Unable to work<br>7. Other
1.7 Have you had any episodes of abdominal pain or heaviness or discomfort in the last 12 months? (If 'no', go to 1.10)<br>1. Yes<br>2. No
1.8 How many days a month?<br>1. One day a month<br>2. Two days a month<br>3. Three days a month<br>4. Four days a month<br>5. 5-10 days per month<br>6. More than 10 days a month
1.9 Have you noticed a reduction in pain after having a bowel movement?<br>1. Yes<br>2. No
1.10 Have you suffered from abdominal swelling in the last 12 months? (If 'no', go to 1.12)<br>1. Yes<br>2. No
1.11 Have you noticed a reduction in swelling after having a bowel movement?<br>1. Yes<br>2. No
1.12 Have you noticed whether the appearance of intestinal disorders is associated with a change in your intestinal regularity? (If you have never complained of intestinal disorders, go to 1.15)<br>1. Yes<br>2. No
1.13 How would you describe these changes?<br>1. Diarrhea<br>2. Constipation<br>3. Mixed bowel movements<br>4. Other
1.14 Do intestinal disorders get worse when you are stressed?<br>1. Yes<br>2. No
1.15 Do you suffer from one or more of the following disorders? (multiple answers possible)<br>1. Anxiety

2. Depression
3. Bipolar Disorder
4. Schizophrenia
5. Thyroid Diseases
6, Diabetes Mellitus
7. Pernicious Anemia (Vitamin B12 Deficiency)
8. Chronic Fatigue
9. Fibromyalgia
10. Chronic Intestinal Inflammatory Diseases
11. Chronic Headache
12. Irritable Bowel Syndrome
13. Celiac Disease
14. Gastroesophageal reflux
15. Other autoimmune diseases
16. Other non-autoimmune diseases

PART 2: SYMPTOMS DUE TO WHEAT INTAKE

2.1 Have you ever noticed a worsening of the symptoms related to Sjögren's disease after eating wheat-containing foods? (If you do not suffer from Sjögren's Syndrome, go to 2.7)
1. Yes
2. No

2.2 Could you describe which symptom(s) you think became worse?
1. Xerostomia
2. Xerophthalmia
3. Xerostomia and xerophthalmia

2.3 Could it indicate a worsening of each symptom?
1. Very slight worsening, but no need to change the therapy or its dosage (VAS 1-3)
2. Worsening which determined the modification of therapy, but you didn't consult a doctor (VAS 4-6)
3. Worsening which led you to consult your doctor (VAS 7-10)

2.4 Have you noticed an improvement in one or more of the symptoms related to Sjögren's disease by eliminating wheat from your diet? (If 'no', go to 2.7)
1. Yes
2. No

2.5 Could you describe which symptom(s) you believe has improved?
1. Xerostomia
2. Xerophthalmia
3. Xerostomia and xerophthalmia

2.6 Could you indicate the improvement for each symptom?
1. Very slight improvement (VAS 1-2)
2. Slight improvement (VAS 3-4)
3. Satisfactory improvement (VAS 5-6)
4. Clear improvement which permitted a reduction in therapy (VAS 7-8)
5. Very clear improvement which led to the disappearance of symptoms and permitted therapy to be suspended (VAS 9-10)

2.7 Have you ever had symptoms (unrelated to Sjögren's disease) after eating wheat-containing foods? (If 'no', go to 3.1)
1. Yes
2. No

2.8 What symptoms did you have after eating wheat-containing foods? (Multiple answers possible)
1. Intestinal bloating
2. Abdominal pain
3. Abdominal heaviness
4. Diarrhea
5. Constipation
6. Lack of energy
7. Belching
8. Flatulence
9. Nausea and/or vomiting
10. Headache
11. Poor motor coordination
12. Numbness or pricking sensation on the skin
13. Anemia
14. Redness of the skin
15. Joint pain
16. Other (specify)

2.9 How often do you suffer from these symptoms after eating wheat-containing foods?
1. Always
2. Often (≥3 days/week)

3. A few days a week (<3 days/week)<br>4. A few times a month (once or more a month)<br>5. A few times a year (less than once a month)<br>6. Less than once a year
2.10 How soon do symptoms appear after ingesting wheat-containing foods?<br>1. Almost immediately (less than an hour)<br>2. 1-6 hours later<br>3. 6-24 hours later<br>4. The next day<br>5. A few days later
2.11 How long do the symptoms last?<br>1. A few minutes<br>2. A few hours<br>3. A few days<br>4. A few weeks<br>5. A few months
2.12 Which product(s)? (multiple answers possible)<br>1. Cereals<br>2. Bread<br>3. Pasta<br>4. Pizza<br>5. Biscuits<br>6. Sweets/Candies<br>7. Other (specify)
2.13 How long have you had a problem with wheat-containing foods? (months)
2.14 Have you ever consulted a doctor/dietician/health care specialist for these disorders?<br>1. Yes<br>2. No
2.15 Specialist consulted (multiple answers possible)<br>1. Gastroenterologist<br>2. General practitioner<br>3. Dietitian<br>4. Other (pharmacist, another medical specialist, specify)<br>1. Allergologist<br>2. Hematologist<br>3. Rheumatologist
2.16 Testing carried out (multiple answers possible)<br>1. Blood test for celiac disease<br>2. Prick test for food allergy<br>3. Esophagogastroduodenoscopy<br>4. Other investigations (specify)<br>1. Abdomen ultrasound examination<br>2. Patch test for nickel allergy<br>3. Genetic (HLA) typing
2.17 Possible explanation (multiple answers possible)<br>1. Celiac Disease<br>2. Wheat Allergy<br>3. Irritable Bowel Syndrome<br>4. No explanation<br>5. Other (specify)<br>1. Non-Celiac Wheat Sensitivity<br>2. Gastritis<br>3. Diverticulosis
2.18 Have you ever followed a wheat-free diet? (If ‘no’, go to 3.1)<br>1. Yes<br>2. No
2.19 Did you have an improvement in your symptoms?<br>1. Yes<br>2. No
2.20 Are you still following a wheat-free diet?<br>1. Yes<br>2. No
2.21 Have you ever consumed products based on ancient grains (e.g. Perciasacchi, Timilia/Tumminia, Senatore Cappelli, etc.)? (If ‘no’, go to 3.1)<br>1. Yes<br>2. No

2.22 Have you experienced the same symptoms that you reported with modern grains when taking ancient grains?<br>1. Yes<br>2. No

**Section 3: self-perceived intolerance to non-wheat/wheat-based foods**
3.1 Have you ever noticed a worsening of the symptoms related to Sjögren’s disease after eating non-wheat/wheat-based foods? (If you do not suffer from Sjögren’s Syndrome, go to 3.7)<br>1. Yes<br>2. No
3.2 Could you describe which symptom(s) you think became worse?<br>1. Xerostomia<br>2. Xerophthalmia<br>3. Xerostomia and xerophthalmia
3.3 Could it indicate a worsening of each symptom?<br>1. Very slight worsening, but no need to change the therapy or its dosage<br>2. Worsening which determined a modification of therapy, but you didn't consult a doctor<br>3. Worsening which led you to consult your doctor
3.4 Have you noticed an improvement in one or more of the symptoms related to Sjögren’s disease by eliminating the foods listed above from your diet? (If ‘no’, go to 3.7)<br>1. Yes<br>2. No
3.5 Could you describe which symptom(s) you believe has improved?<br>1. Xerostomia<br>2. Xerophthalmia<br>3. Xerostomia and xerophthalmia
3.6 Could you indicate the improvement for each symptom?<br>1. Very slight improvement<br>2. Slight improvement<br>3. Satisfactory improvement<br>4. Clear improvement which permitted a reduction in therapy<br>5. Very clear improvement which led to the disappearance of symptoms and permitted therapy to be suspended
3.7 Have you ever had symptoms (unrelated to Sjögren’s disease) after eating non-wheat-based foods? (If ‘no’, end of questionnaire)<br>1. Yes<br>2. No
3.8 Can you indicate which food(s)? (Specify)
3.9 Other symptoms after intake of the foods listed above (multiple responses possible)<br>1. Intestinal bloating<br>2. Abdominal pain<br>3. Abdominal heaviness<br>4. Diarrhea<br>5. Constipation<br>6. Asthenia<br>7. Belching<br>8. Flatulence<br>9. Nausea and/or vomiting<br>10. Headache<br>11. Poor motor coordination<br>12. Numbness or sensation<br>13. Anemia<br>14. Redness of the skin<br>15. Joint pain<br>16. Other (specify)
3.10 How often do you suffer from these symptoms after eating the foods listed above?<br>1. Always<br>2. Often (≥3 days/week)<br>3. A few days a week (<3 days/week)<br>4. A few times a month (once or more a month)<br>5. A few times a year (less than once a month)<br>6. Less than once a year
3.11 How soon after eating the foods listed above do symptoms appear? (n, %)<br>1. Almost immediately (less than an hour)<br>2. 1-6 hours later<br>3. 6-24 hours later<br>4. The next day<br>5. A few days later

3.12 How long do the symptoms last?<br>1. A few minutes<br>2. A few hours<br>3. A few days<br>4. A few weeks<br>5. A few months
3.13 How long have you had this problem with the foods listed above?
3.14 Have you ever consulted a doctor/dietician/health care specialist for these disorders?<br>1. Yes<br>2. No
3.15 Specialist consulted (multiple answers possible)<br>1. Gastroenterologist<br>2. Family doctor<br>3. Dietitian<br>4. Other (pharmacist, another medical specialist, specify)<br>1. Allergologist<br>2. Hematologist<br>3. Rheumatologist
3.16 Testing carried out (multiple answers possible)<br>1. Patch test for nickel allergy<br>2. Prick test for food allergy<br>3. Lactose breath test<br>4. Other investigations for food intolerance (specify)<br>1. Esophagogastroduodenoscopy<br>2. Search for specific IgE
3.17 Possible explanation (multiple answers possible) (n, %)<br>1. Nickel allergy<br>2. Food Allergy<br>3. Lactose Intolerance<br>4. Irritable Bowel Syndrome<br>5. No explanation<br>6. Other (Specify)

VAS: Visual Analogue Scale
