# Supplementary material for: Self-Reported Non-Celiac Wheat Sensitivity and Other Food Sensitivities in Patients with Primary Sjögren’s Syndrome
Source: Nutrients. 2025 Oct 8;17(19):3172. doi: 10.3390/nu17193172 (PMC12525645; doi:10.3390/nu17193172)
Supplement: Supplementary file 1 [file nutrients-17-03172-s001.zip › Supplementary File S2 - Tables - 03_10_2025.pdf]

Supplemental Table S1. Clinical, immunological and therapeutic features of patients with pSS.

	**Patients n=82**
ESSPRI (mean±SD)	5.6±0.6
ESSDAI [median (min-max)]	2 (0-8)
ESSDAI systemic disease activity (n, %)	
Low (ESSDAI <5)	56 (68.3)
Moderate (ESSDAI 5-13)	26 (31.7)
High (ESSDAI ≥14)	0 (0.0)
pSS clinical manifestations (n, %)	
Xerostomia	8 (9.8)
Xerophthalmia	13 (15.9)
Xerostomia and xerophthalmia	61 (74.4)
ANA positivity (n, %)	61 (74.4)
ANA (titer) (n, %)	N 61
1:80	1 (1.6)
1:160	35 (57.4)
1:320	5 (8.2)
1:640	4 (6.6)
1:1320	14 (23)
>1:1320	2 (3.3)
ANA (pattern) (n, %)	
AC-1 (Nuclear Homogeneous)	9 (14.8)
AC-2 (Nuclear Dense Fine Speckled)	2 (3.28)
AC-4 (Nuclear Fine Speckled)	49 (80.3)
AC-6 (Multiple Nuclear Dots)	1 (1.6)
AC-8 (Homogeneous Nucleolar)	0 (0.0)
AC-26 (NuMA-like)	0 (0.0)
anti-SSA positivity (n, %)	44 (53.7)
anti-SSB positivity (n, %)	12 (14.6)
RF (n, %)	30 (36.6)
HypoC3 (<80 mg/dl) (n, %)	8 (9.8)
Value (mg/dl) (mean±SD)	107.9±25.9
HypoC4 (<15 mg/dl) (n, %)	8 (9.8)
Value (mg/dl) (mean±SD)	22.6±11
Hypergammaglobulinemia (>1.5 g/dl) (n, %)	25 (30.55)
Value (mg/dl) (mean±SD)	1.5±0.5
Monoclonal component (n, %)	8 (9.8)
Monoclonal component characteristics (n, %)	N 8
IgG Kappa	6 (75.0)
IgG Lambda	1 (12.5)
IgA Kappa	1 (12.5)
Xerostomia treatment (n, %)	N 69
Mechanical or gustatory stimulation techniques	58 (84.1)
Artificial saliva	11 (15.9)
Xerophthalmia treatment (n, %)	N 74
Lubricant ointments	68 (91.9)
Topical cyclosporine	6 (8.1)

ANA=Anti-Nuclear Antibodies; C3=Complement Component 3; C4=Complement Component 4; ESSDAI=European League Against Rheumatism (EULAR) Sjögren's Syndrome Disease Activity Index; ESSPRI=EULAR Sjögren's Syndrome Patient Reported Index; pSS=primary Sjögren's Syndrome; RF=Rheumatoid Factor; SSA=Sjögren's-syndrome-related antigen A; SSB=Sjögren's-syndrome-related antigen B.

Supplemental Table S2. List, in alphabetical order, of non-wheat-containing foods referred by pSS patients and controls as causing symptoms (unrelated to pSS) (Multiple foods may be indicated per patient).

	pSS (n=35)	Controls (n=21)	*P*
**Milk and dairy products**	24 (68.6)	9 (42.9)	NS
Aged dairy products	6 (25.0%)	6 (66.7)	NS
Fresh dairy products	13 (54.2%)	3 (33.3)	NS
Milk	24 (100.0%)	9 (100.0)	NS
**Other foods**	25 (71.4)	15 (71.4)	NS
Artichokes	4 (16.0)	0 (0.0)	NS
Blue/Oily/Fatty fish	4 (16.0)	3 (20.0)	NS
Broccoli	1 (4.0)	0 (0.0)	NS
Broth	1 (4.0)	0 (0.0)	NS
Carbonated drinks	1 (4.0)	0 (0.0)	NS
Chicken	1 (4.0)	3 (20.0)	NS
Chocolate	2 (8.0)	0 (0.0)	NS
Citrus fruits	3 (12.0)	0 (0.0)	NS
Coffee	1 (4.0)	0 (0.0)	NS
Eggplant	3 (12.0)	0 (0.0)	NS
Fennel seeds	0 (0.0)	1 (6.7)	NS
French fries	4 (16.0)	0 (0.0)	NS
Fructose	1 (4.0)	0 (0.0)	NS
Garlic	1 (4.0)	0 (0.0)	NS
Grapes	1 (4.0)	0 (0.0)	NS
Legumes	6 (24.0)	0 (0.0)	NS
Mayonnaise	2 (8.0)	0 (0.0)	NS
Mollusks	2 (8.0)	0 (0.0)	NS
Mushrooms	1 (4.0)	0 (0.0)	NS
Pears	1 (4.0)	0 (0.0)	NS
Peppers	3 (12.0)	0 (0.0)	NS
Pineapple	1 (4.0)	0 (0.0)	NS
Plums	1 (4.0)	0 (0.0)	NS
Pork	1 (4.0)	0 (0.0)	NS
Potatoes	1 (4.0)	0 (0.0)	NS
Rice	1 (4.0)	0 (0.0)	NS
Sausages	2 (8.0)	0 (0.0)	NS
Crustaceans	2 (8.0)	0 (0.0)	NS
Strawberries	1 (4.0)	0 (0.0)	NS
Tomato	11 (44.0)	3 (20.0)	NS
Tomato sauce	2 (8.0)	0 (0.0)	NS
Unripe fruit	1 (4.0)	0 (0.0)	NS
Vegetables (others)	6 (24.0)	3 (20.0)	NS

NS=Not Significant; pSS=Primary Sjögren's Syndrome.

Supplemental Table S3. Demographic features of the pSS patients enrolled in the study with or without self-reported NCWS.

	**pSS patients self-reporting NCWS (n=39)**	**pSS patients non self-reporting NCWS (n=43)**	***P***
Age (years) (mean±SD)	60.4±10.8	64.3±11.1	NS
Gender (n, %)			
1. Male	1 (2.6)	3 (7.0)	NS
2. Female	38 (97.4)	40 (93.0)	NS
Ethnicity (n, %)			
1. Caucasian	39 (100.0)	43 (100.0)	NS
2. African	(0.0)	(0.0)	NS
3. Asian	(0.0)	(0.0)	NS
4. Middle Eastern	(0.0)	(0.0)	NS
Marital status (n, %)			
1. Single	7 (17.9)	4 (9.3)	NS
2. Married	27 (69.2)	31 (72.1)	NS
3. Divorced	1 (2.6)	2 (4.7)	NS
4. Widowed	4 (10.3)	6 (14)	NS
Qualification (n, %)			
1. None	3 (7.7)	0 (0.0)	NS
2. Elementary school diploma	1 (2.6)	7 (16.3)	NS
3. Middle school diploma	16 (39)	16 (37.2)	NS
4. High school diploma	14 (35.4)	15 (34.9)	NS
5. University degree	5 (12.2)	5 (11.6)	NS
Employment status (n, %)			
1. Employee	10 (25.6)	6 (14.0)	NS
2. Freelance/Professional	2 (25.1)	3 (7.0)	NS
3. Laborer/unskilled worker,	0 (0.0)	2 (4.7)	NS
craftsperson	22 (56.4)	21 (48.8)	NS
4. Unemployed	1 (2.6)	1 (2.3)	NS
5. Looking for work	0 (0.0)	0 (0.0)	NS
6. Unable to work	4 (10.3)	10 (23.3)	NS
7. Other			

NCWS=Non-Celiac Wheat Sensitivity; NS=Not Significant; pSS=primary Sjögren's Syndrome; SD=Standard Deviation.

Supplemental Table S4. Clinical, immunological and therapeutic features in pSS patients with or without self-reported NCWS.

	pSS patients self-reporting NCWS (n=39)	pSS patients non-self-reporting NCWS (n=43)	*P*
ESSPRI (mean±SD)	5.8±1.6	5.4±1.7	NS
ESSDAI [median (min-max)]	2 (0-7)	2 (0-8)	NS
ESSDAI systemic disease activity (n, %)			
Low (ESSDAI <5)	27 (69.2)	29 (67.4)	NS
Moderate (ESSDAI 5-13)	12 (30.8)	14 (32.6)	NS
High (ESSDAI ≥14)	0 (0.0)	0 (0.0)	NA
pSS clinical manifestations (n, %)			
Xerostomia	3 (7.7)	5 (11.6)	NS
Xerophthalmia	5 (12.8)	8 (18.6)	NS
Xerostomia and xerophthalmia	31 (79.5)	30 (69.8)	NS
ANA positivity (n, %)	28 (73.7)	33 (78.6)	NS
ANA (titer) (n, %)	N=28	N=33	
1:80	1 (3.7)	0 (0.0)	NS
1:160	13 (46.4)	22 (66.7)	NS
1:320	1 (3.6)	4 (12.1)	NS
1:640	2 (7.1)	2 (6.1)	NS
1:1320	9 (3.2)	5 (15.2)	NS
>1:1320	2 (7.1)	0 (0.0)	NS
ANA (pattern) (n, %)			
AC-1 (Nuclear Homogeneous)	5 (15.2)	4 (11.4)	NS
AC-2 (Nuclear Dense Fine Speckled)	1 (3.0)	1 (2.9)	NS
AC-4 (Nuclear Fine Speckled)	22 (66.7)	27 (77.1)	NS
AC-6 (Multiple Nuclear Dots)	0 (0.0)	0 (0.0)	NS
AC-8 (Homogeneous Nucleolar)	0 (0.0)	1 (2.9)	NS
AC-26 (NuMA-like)	0 (0.0)	0 (0.0)	NS
anti-SSA positivity (n, %)	22 (56.4)	22 (51.2)	NS
anti-SSB positivity (n, %)	6 (15.4)	6 (14.0)	NS
RF (n, %)	15 (38.5)	15 (34.9)	NS
HypoC3 (<80 mg/dl) (n, %)	4 (10.5)	4 (9.5)	NS
Value (mg/dl) (mean±SD)	109±15.6	106±17.8	NS
HypoC4 (<15 mg/dl) (n, %)	3 (7.9)	5 (11.9)	NS
Value (mg/dl) (mean±SD)	25.7±12.3	18.4±7.5	NS
Hypergammaglobulinemia (>1.5 g/dl) (n, %)	14 (35.9)	11 (25.6)	NS
Value (mg/dl) (mean±SD)	1.6±0.65	1.4±0.44	NS
Monoclonal component (n, %)	6 (15.4)	2 (4.7)	NS
Monoclonal component characteristics (n, %)			
IgG Kappa	4 (66.7)	2 (100)	NS
IgG Lambda	1 (16.7)	0 (0.0)	NS
IgA Kappa	1 (16.7)	0 (0.0)	NS
Xerostomia treatment (n, %)	N 34	N 35	
Mechanical or gustatory stimulation techniques	28 (82.4)	30 (85.7)	NS
Artificial saliva	6 (17.6)	5 (14.3)	NS
Xerophthalmia treatment (n, %)	N 36	N 38	
Lubricant ointments	34 (94.4)	34 (89.5)	NS
Topical cyclosporine	2 (5.6)	4 (10.5)	NS
SRMI (n, %)	15 (39.5)	5 (11.9)	0.01
MFS (n, %)	25 (65.7)	10 (23.8)	0.0004

ANA=Anti-Nuclear Antibodies; C3=Complement Component 3; C4=Complement Component 4; ESSDAI=European League Against Rheumatism (EULAR) Sjögren's Syndrome Disease Activity Index; ESSPRI=EULAR Sjögren's Syndrome Patient Reported Index; MFS=Multiple Food Sensitivity; NA=Not Applicable; pSS=primary Sjögren's Syndrome; RF=Rheumatoid Factor; SRMI=Self-Reported Milk Intolerance; SSA=Sjögren's-syndrome-related antigen A; SSB=Sjögren's-syndrome-related antigen B.
