# Supplementary figures and images for: Self-Reported Non-Celiac Wheat Sensitivity and Other Food Sensitivities in Patients with Primary Sjögren’s Syndrome
Source: Nutrients. 2025 Oct 8;17(19):3172. doi: 10.3390/nu17193172 (PMC12525645; doi:10.3390/nu17193172)

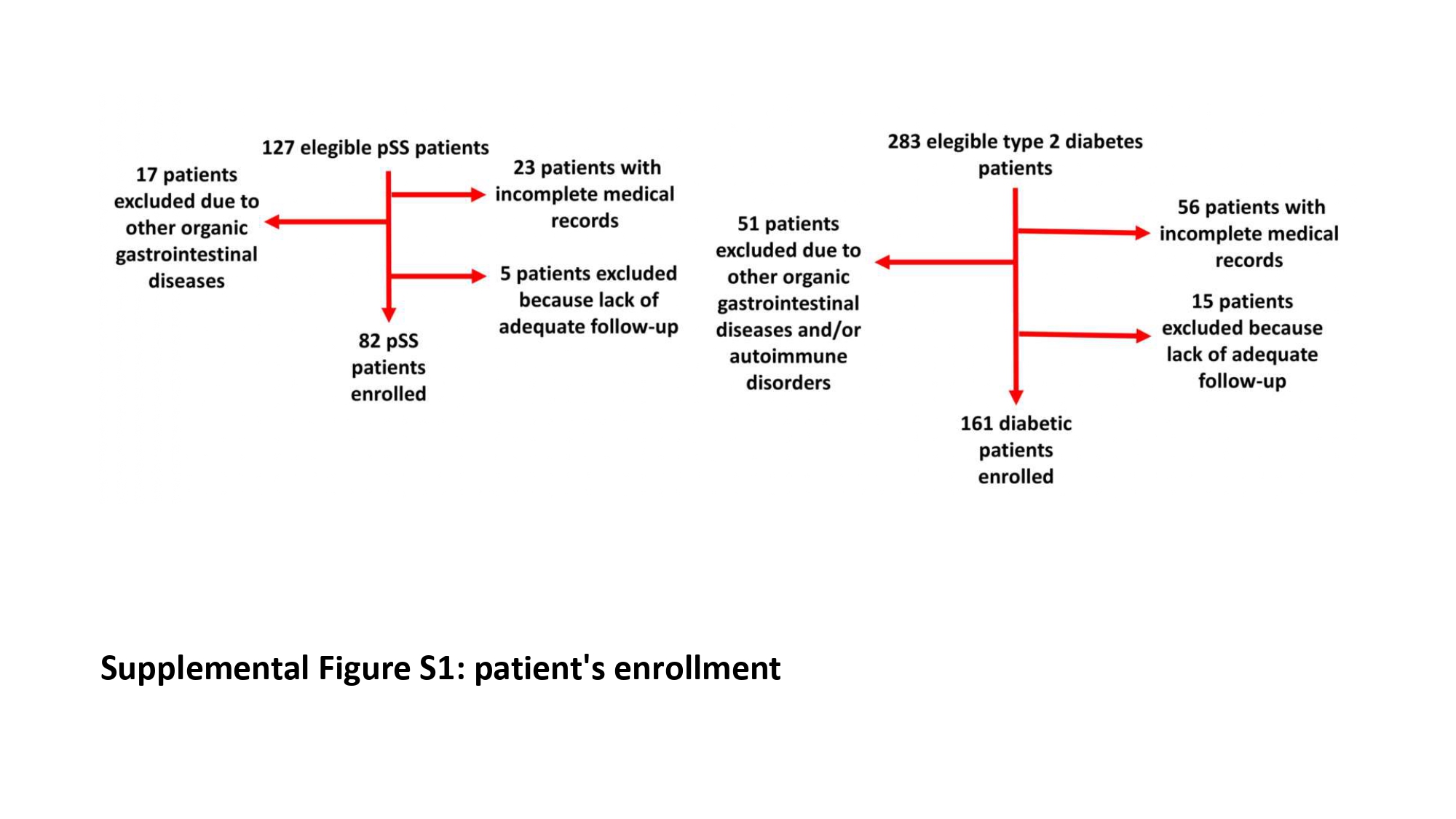

Supplement: Supplementary file 1 [file nutrients-17-03172-s001.zip › Supplementary Figure S1 - 11_09_2025.jpg]
